# Side Effects of Psychotropic Medications Experienced by a Community Sample of People Living With Severe and Persistent Mental Illness

**DOI:** 10.1111/hex.70122

**Published:** 2024-12-11

**Authors:** Jack C. Collins, Amanda J. Wheeler, Sara S. McMillan, Jie Hu, Sarira El‐Den, Helena Roennfeldt, Claire L. O'Reilly

**Affiliations:** ^1^ The University of Sydney School of Pharmacy, Faculty of Medicine and Health The University of Sydney Sydney Australia; ^2^ Menzies Health Institute Queensland Griffith University Nathan Australia; ^3^ School of Pharmacy and Medical Sciences Griffith University Gold Coast Australia; ^4^ School of Pharmacy, Faculty of Health and Behavioural Sciences University of Auckland Auckland New Zealand; ^5^ Centre for Mental Health Griffith University Nathan Australia

**Keywords:** drug side effects, mental illness, psychotropic drugs, severe mental disorders, treatment burden

## Abstract

**Background:**

Psychotropic medications are a common treatment modality for people living with severe and persistent mental illness (SPMI). While effective in reducing relapse and hospitalisation, psychotropic medications cause numerous side effects, varying in nature and severity. Identification and management of side effects is crucial in the ongoing management of SPMI.

**Objective:**

To characterise the side effects of psychotropic medications, experienced by a sample of consumers living with SPMI, using a validated tool.

**Setting and Participants:**

Consumers with SPMI living in the community were recruited from all 25 community pharmacies across four Australian regions, which were allocated to the intervention arm of the Bridging the Gap between Physical and Mental Illness (*PharMIbridge*) randomised controlled trial (RCT).

**Main Outcome Measures:**

Responses to the *My Medicines & Me Questionnaire* (*M3Q)*.

**Results:**

Consumers (*n* = 156) most frequently reported side effects in the categories of sleep‐related side effects (80.8%, *n* = 126), mood‐related side effects (75.6%, *n* = 118) and weight and appetite changes (60.3%, *n* = 107). Daytime somnolence was the most reported individual side effect (68.6%, *n* = 107). Mood‐related side effects were ranked as the most bothersome, followed by sleep‐related side effects and weight and appetite changes. More than one‐quarter (29.5%, *n* = 46) of consumers reported choosing not to take their medications due to side effects. Consumers more frequently told family and friends about the side effects rather than healthcare professionals.

**Conclusions:**

An overwhelming majority of consumers experienced at least one side effect attributed to their psychotropic medication, with many experiencing multiple. These findings highlight the critical need to regularly engage with consumers to discuss, identify and manage side effects to treatment burden, reduce risk of non‐adherence and improve their treatment experience.

**Patient or Public Contribution:**

The *PharMIbridge* RCT included a training programme and intervention service that was co‐designed and co‐delivered with people with lived experience of mental illness. The research team, expert advisory panel and mentors who supported the delivery and implementation of the training and intervention included participants who have lived experience of mental illness or caring for someone with mental illness.

**Trial Registration:**

ANZCTR12620000577910.

## 1 Introduction

Severe and persistent mental illness (SPMI) is a cluster of conditions that are typically characterised as chronic and recurring and which can negatively impact the daily lives of those living with these conditions [1]. Schizophrenia and related disorders, bipolar disorder, moderate to severe depression and moderate to severe anxiety, among other conditions, are classified as SPMI. Although quantifying the number of people living with SPMI is challenging due to a lack of a consensus definition for SPMI [1], it is estimated, for example, that approximately 300,000 people in Australia (approximately 1.2% of the population) live with SPMI [2].

Due to the chronic, recurrent and potentially debilitating nature of SPMI, treatment with psychotropic medications for a prolonged duration (often life‐long) is often recommended [3, 4]. For example, continuous treatment with antipsychotic medications in people living with schizophrenia has been associated with reduced risk of relapse than in those who take medications intermittently, reduce doses or cease medications altogether [5, 6]. Although psychotropic medications can have significant benefits for people living with SPMI, including improved morbidity and mortality rates [7], these are counterbalanced with considerable side effects which may be have a considerable impact on consumers [8, 9].

Side effects, such as weight gain, perceived impairment of cognition and sedation, may be so bothersome that people may choose to stop taking their medications, putting them at an increased risk of relapse and hospitalisation [10, 11, 12, 13]. Certain psychotropic medications, such as antipsychotics, may also result in cardiometabolic side effects such as weight gain, hypertension, hypercholesterolaemia and hyperglycaemia [14, 15]. This is of particular concern in a population which already experiences a significant gap in life expectancy due to suboptimally managed physical health conditions [16, 17]. Furthermore, psychotropic polypharmacy (the concurrent use of multiple psychotropic medications) is common among people living with SPMI which increases the risk of experiencing side effects [18, 19, 20], and polypharmacy with both psychotropic and non‐psychotropic medications also presents risk of clinically significant drug–drug interactions [21, 22].

Given the potential significance of side effects, it is critical that members of the healthcare team screen for the presence of these side effects and that consumers are afforded the opportunity to express any concerns regarding their psychotropic medications, noting that they are likely to underreport the presence of side effects unless asked [23]. Furthermore, the post‐market reports of both incidence and types of side effects likely vary considerably from trial and drug registration data which are often captured over shorter time periods in relatively homogenous populations [8], indicating a need to capture this information from consumers. Considering shortfalls in screening for the presence of side effects in clinical practice [24, 25], a number of tools have been created to assist members of the care team to screen for side effects and engage in conversations with consumers [26, 27]. Screening for side effects, such as through using validated tools, also enables consumers to report side effects that they determine to be bothersome or a priority [26], which may not necessarily align with the priorities of the healthcare team [28].

As medication experts, pharmacists are well‐placed to engage with consumers about their medications, including the presence of any side effects. Evidence shows pharmacists can identify side effects during medication supply and reviews and provide education to consumers about medication side effects, potentially improving adherence [29, 30, 31, 32]. This presents the opportunity for pharmacists to use a validated tool to screen for side effects of psychotropic medications and is supported by calls for more field studies on the side effects of a range of psychotropic medications and the need for tools that allow consumers to adequately express their subjective lived experience of side effects [26, 33]. Consequently, this study aimed to characterise the lived experience of side effects of psychotropic medications reported by a community‐residing sample of consumers living with SPMI during a medication review with community pharmacists using a validated tool designed for consumers.

## 2 Methods

### 2.1 Ethical Approval

The study from which this data is obtained received approval from the Griffith University Human Research Ethics Committee (HREC/2019/473).

### 2.2 Consumer Participants and Setting

People living with SPMI were recruited via community pharmacies as part of the Bridging the Gap between Physical and Mental Illness (*PharMIbridge*) cluster randomised controlled trial (RCT) between September 2020 to February 2021. The detailed protocol [34] and trial registration for the RCT [35] are previously published and provide further detail regarding the rationale and design of the RCT. For the purpose of the RCT, consumers were eligible to participate if they were (1) aged 16 years or older and living in the community and (2) had at least a 6‐month history of use of an antipsychotic or mood stabiliser medication for the treatment of self‐reported SPMI and (3) had complex medication needs (e.g., managing multiple medications) or unmanaged physical health issues (e.g., weight gain). Consumers could participate in the RCT either by expressing interest to a staff member after seeing recruitment collateral in the pharmacy or by being directly approached by trained pharmacy staff.

### 2.3 Community Pharmacies Sites and Training

Community pharmacies (*n* = 51) were recruited across four Australian regions (Australian Capital Territory, Hunter New England, northern Sydney and regional Victoria), screened for eligibility and randomised to the Intervention Group (IG; *n* = 25) or Comparator Group (CG; *n* = 26). The sample of consumers included in this study were recruited from pharmacies in the IG and completed the intervention service (see Section 2.4). Pharmacy staff from pharmacies randomised to the IG attended a 2‐day face‐to‐face training workshop which included Blended Mental Health First Aid for Pharmacy training [36], role‐play scenarios and four modules that addressed the mind‐body interface, complex psychotropic medication use, supporting adherence and physical wellbeing; and communication, motivational interviewing and goal setting. Further descriptions and evaluations of the training are available elsewhere [37, 38, 39].

### 2.4 Data Collection

After being screened for eligibility and providing informed consent to participate in the RCT, consumer participants completed an electronic baseline survey to record demographic details and responses to a number of validated instruments which explored treatment burden, quality of life and psychological distress, among others (see RCT protocol [34] for further information). Following baseline data collection, pharmacists worked with consumer participants to complete the first component of the intervention, the *Initial Health Review*. The *Initial Health Review* could take place on the same day as initial baseline data collection or at a later date which was mutually agreed upon by the pharmacist and consumer.

During the *Initial Health Review* pharmacists and consumers reviewed key findings from responses to the baseline survey and completed additional validated instruments. All participants were asked to complete the *My Medicines and Me Questionnaire* (*M3Q*) [40] (Section 2.4.1). After completing these instruments, pharmacists completed reconciliation and review of all current psychotropic and non‐psychotropic medications. Medication lists were generated using dispensing software data and consumer self‐reports. Pharmacists worked with consumers to identify key areas of concern regarding medications, physical health, mental health or other relevant concerns, set goals together with consumers to address these issues and worked with consumers to develop an individualised support plan to assist consumers to work on their goals over the 6‐month intervention period. Data entry during the *Initial Health Review* was completed electronically by pharmacists using modules specifically designed and built for the RCT which were integrated into an existing pharmacy professional practice software, GuildCare.

#### 2.4.1 M3Q

The *M3Q* [40] is a simple tool designed to help consumers self‐report subjective and objective side effects of psychotropic medications experienced in the previous 4 weeks. The side effects are broadly classified into 11 categories, primarily based on body system or function. Consumers are then prompted to rank the three most bothersome side effects and complete additional questions regarding medications suspected to be contributing to the side effect, frequency of experiencing the side effect and how long it lasts, its impact on daily living, and if other people are aware that the side effect is being experienced. The *M3Q* ends with questions regarding the impact of side effects on intended or actual adherence to medications, perceived benefits of medication use and any further information. The instrument has been tested for content, construct and criterion validity, reliability (Cronbach's *α* = 0.928) and usability with mental health consumers [41, 42, 43]. In this study, verbal responses to the *M3Q* from consumer participants were documented by pharmacists.

### 2.5 Quantitative Data Analysis

Raw data from the baseline survey and *Initial Health Review* were exported to Microsoft Excel (Microsoft Corp, Redmond, USA) for preliminary data cleaning before being imported into IBM SPSS Statistics V28.0 (IBM Corp, Armonk, USA) for descriptive analysis. Brief and structured text responses, such as the names of medications and unlisted side effects, were cleaned and re‐coded for consistency by a single author (J.C.C.). Continuous data were tested for normality, and reported as means and standard deviations or medians and interquartile ranges, where appropriate. Nominal data were reported as frequencies and proportions. To identify the most bothersome side effects, a rank sum was generated whereby a value of 1–3 was assigned to each report of the third‐most, second‐most and most bothersome side effect, respectively.

### 2.6 Qualitative Data Analysis

Open‐ended responses to the items regarding why medications were stopped by consumer participants and the perceived benefits of using the medications from the *M3Q* were exported as raw data to Microsoft Excel (Microsoft Corp, Redmond, USA) for analysis. Due to the brevity of free‐text response data, a content analysis [44] was performed by a single author (C.L.O.R., a pharmacist and mental health researcher). Following familiarisation with the data, codes were developed which represented key characteristics of responses. Codes with commonalities were then clustered into higher order categories. Preliminary codes and categories were reviewed and discussed between two researchers (C.L.O.R. and J.C.C.) to finalise the codes and categories.

## 3 Results

### 3.1 Consumer Demographics and Reported Medication Use

The *Initial Health Review* was completed with 156 consumer participants. Consumer participants had a mean age of 48.1 years (SD: ±12.6 years), slightly more than half identified as female (53.8%, *n* = 84) and resided in urban/metropolitan areas (55.8%, *n* = 87). Additional demographics of consumer participants are shown in Table 1. Medications for mental health conditions were most often (45.5%, *n* = 71) prescribed by a general practitioner (GP) or multiple prescriber types (31.4%, *n* = 49). A majority of consumers (90.4%, *n* = 141) were prescribed more than one psychotropic medication, most commonly an antipsychotic (82.7%, *n* = 129). Almost one‐third of the sample were prescribed antidepressants (30.3%, *n* = 128) and/or benzodiazepines (9.0%, *n* = 38) in addition to antipsychotics and/or mood stabilisers. A total of 423 individual instances of psychotropic medications were recorded for the 156 participants. The most common individual medications recorded were quetiapine (*n* = 62), olanzapine (*n* = 40) and lamotrigine (*n* = 33). Further detail regarding the psychotropic medications used by consumer participants is shown in Tables 2 and 3.

**Table 1:** Participant demographics (*n* = 156).

Characteristic	*n* (%)
Age (years), mean (SD)	48.1 (12.6)
Age group in years	
≤ 24	5 (3.2)
25–34	24 (15.4)
35–44	33 (21.2)
45–54	44 (28.2)
55–64	37 (23.7)
65–74	10 (6.4)
≥ 75	3 (1.9)
Gender	
Female	84 (53.8)
Male	70 (44.9)
Not specified	2 (1.3)
Born in Australia	144 (92.3)
Speak English at home	153 (98.1)
Location^a^	
Urban/metropolitan (MMM 1–2)	87 (55.8)
Non‐metropolitan (MMM 3–7)	69 (44.2)
Self‐reported mental illness^b^	
Moderate‐severe depression	91 (58.3)
Moderate‐severe anxiety disorder	82 (52.6)
Bipolar disorder	71 (45.5)
Schizophrenia/schizoaffective disorder	39 (25.0)
Personality disorder	20 (12.8)
Substance use disorder	6 (3.8)
Other	8 (5.1)

^a^ Location was determined using the Modified Monash Model [45] classification for the postcode of the community pharmacy.

^b^ Participants could report more than one mental illness.

**Table 2:** Overview of prescribed psychotropic medications (*n* = 156).

Characteristic	*n* (%)
Self‐reported prescriber for psychotropic medications	
General practitioner only	71 (45.5)
Multiple prescribers	49 (31.4)
Psychiatrist only	31 (19.9)
Nurse practitioner only	1 (0.6)
Not reported	4 (2.6)
Reported number of psychotropic medications^a^	
1	15 (9.6)
2	59 (37.8)
3	50 (32.1)
4	22 (14.1)
5	8 (5.1)
6	2 (1.3)
Number of classes of psychotropic medications	
1	19 (12.2)
2	80 (51.3)
3	53 (34.0)
4	4 (2.6)
Psychotropic medication classes^b^	
Antipsychotics	129 (82.7)
Antidepressants	111 (71.2)
Mood stabilisers	78 (50.0)
Benzodiazepines	36 (23.1)

^a^ Participants may have used more than one strength/formulation of a medication.

^b^ Participants may have more than one medication prescribed in each class.

**Table 3:** Psychotropic medications (*n* = 423) used by 156 consumer participants.

Medication	*n* (%^a^, ^b^)
Antipsychotics	160 (37.8)
Amisulpride	1 (0.2)
Aripiprazole	23 (5.4)
Asenapine	1 (0.2)
Brexpiprazole	2 (0.5)
Chlorpromazine	2 (0.5)
Clozapine	12 (2.8)
Haloperidol	2 (0.5)
Lurasidone	8 (1.9)
Olanzapine	40 (9.5)
Paliperidone	3 (0.7)
Risperidone	2 (0.5)
Quetiapine	62 (14.7)
Ziprasidone	1 (0.2)
Zuclopenthixol	1 (0.2)
Antidepressants	128 (30.3)
Agomelatine	2 (0.5)
Amitriptyline	7 (1.7)
Citalopram	5 (1.2)
Clomipramine	1 (0.2)
Desvenlafaxine	17 (4.0)
Dosulepin	1 (0.2)
Doxepin	2 (0.5)
Duloxetine	6 (1.4)
Escitalopram	15 (3.5)
Fluoxetine	12 (2.8)
Fluvoxamine	1 (0.2)
Mirtazapine	22 (5.2)
Nortriptyline	4 (0.9)
Paroxetine	2 (0.5)
Reboxetine	1 (0.2)
Sertraline	10 (2.4)
Tranylcypromine	1 (0.2)
Venlafaxine	17 (4.0)
Vortioxetine	2 (0.5)
Mood stabilisers	97 (22.9)
Carbamazepine	1 (0.2)
Lamotrigine	33 (7.8)
Lithium	31 (7.3)
Oxcarbazepine	1 (0.2)
Topiramate	6 (1.4)
Valproate	25 (5.9)
Benzodiazepines	38 (9.0)
Alprazolam	1 (0.2)
Clonazepam	3 (0.4)
Diazepam	24 (5.7)
Lorazepam	1 (0.2)
Nitrazepam	2 (0.5)
Temazepam	7 (1.7)

^a^ Proportion of all reported psychotropic medications (*n* = 423).

^b^ Does not total 100% due to rounding.

### 3.2 Common Reported Side Effects

The frequencies of side effects reported in the *M3Q* responses are shown in Table 4. The top three most reported categories of side effects, with consumer participants reporting at least one side effect in the category, were ‘sleep‐related issues’ (80.8%, *n* = 126), ‘mood’ (75.6%, *n* = 118) and ‘weight and appetite changes’ (60.3%, *n* = 94). Feeling tired during the day was the most‐reported individual side effect, with more than two‐thirds (68.6%, *n* = 107) of participants reporting experiencing this. This was followed by difficulties waking up fresh in the morning (57.7%, *n* = 90). Mood‐related side effects such as feeling anxious (58.3%, *n* = 91), agitated (51.3%, *n* = 80) or sad (51.3%, *n* = 80) were also commonly reported. Just over 40% of participants (*n* = 63) reported gaining weight as a result of their medications. Almost one‐fifth (18.6%, *n* = 29) of participants reported a side effect that was not included in the standard *M3Q* options. Fourteen participants (9.0%) did not have a response to any of the side effects documented and were classified as missing data for this question.

**Table 4:** Side effects reported in *M3Q* responses (*n* = 156).

Side effect^a^	*n* (%)
Felt tired during the day	107 (68.6)
Felt anxious	91 (58.3)
Had difficulties waking up fresh in the morning	90 (57.7)
Felt agitated	80 (51.3)
Felt sad	80 (51.3)
Gained weight	63 (40.4)
Felt weak	56 (35.9)
Lost interest in enjoyable things	53 (34.0)
Had difficulty staying awake during the day	47 (30.1)
Been more thirsty than usual	47 (30.1)
Less interested in sex	45 (28.8)
Arms or legs been shaky	44 (28.2)
Felt drugged or like a zombie	43 (27.6)
Needed to go to the toilet often	42 (26.9)
Restless legs	42 (26.9)
Felt more hungry than usual	41 (26.3)
Found that words do not come out clearly	40 (25.6)
Vision been blurry	36 (23.1)
Sweating more than usual	35 (22.4)
Eyes felt dry and gritty	28 (17.9)
Stools been hard or difficult to pass	27 (17.3)
Found it difficult to enjoy sex	26 (16.7)
Found it difficult to swallow	24 (15.4)
Have experienced fits or jerks	22 (14.1)
Have diabetes	22 (14.1)
Been unable to reach orgasm	19 (12.2)
Have been told that blood sugar levels are high	19 (12.2)
Think some food tastes different or odd	16 (10.3)
Skin been more sensitive to the sun	16 (10.3)
Noticed a change in blood sugar levels	10 (6.4)
Noticed areas of darker skin	9 (5.8)
Areas around nipples sore or swollen	4 (2.6)
Any other side effects not listed^b^	29 (18.6)
No side effects from list selected^c^	14 (9.0)

^a^ Individual side effects—see *M3Q* tool for higher order categories.

^b^ Xerostomia and nausea were the most reported ‘other’ side effects.

^c^ Unable to determine if missing data or participant not experiencing any side effects.

### 3.3 Most Bothersome Side Effects and Intention to Cease Medications

Of 156 consumer participants who completed the *Initial Health Review*, 95.5% (*n* = 149) ranked at least one side effect in the ranking section of the *M3Q*. The remaining participants did not conduct any ranking and were classified as missing data for this question. The rank sum identified that the three most‐bothersome categories of side effects were ‘mood’, ‘sleep‐related issues’ and ‘weight and appetite changes’. The full ranking of categories of side effects is shown in Table 5. When asked about how frequently the most bothersome side effect was experienced, the majority (73.7%, *n* = 115) reported that they experienced the side effect on a daily basis. Twenty‐two participants (14.1%) experienced the side effect weekly, and nine participants (5.8%) experienced the side effect monthly.

**Table 5:** Ranking of side effect categories considered most bothersome (*n* = 156).

Side effect category	Ranking	Frequency^a^ *n* (%)
Mood	1	118 (75.6)
Sleep‐related issues	2	126 (80.8)
Weight and appetite changes	3	94 (60.3)
General health	4	86 (55.1)
Bowel and bladder habits	5	61 (39.1)
Uncontrollable face and body movements	6	75 (48.1)
Diabetes	7	31 (19.9)
Sexual health	8	51 (32.7)
Oral problems	9	49 (31.4)
Visual problems	10	48 (30.8)
Skin changes	11	20 (12.8)
No ranking reported	—	7 (4.5)

a Number of participants reporting at least one of the side effects listed in this category, as participants could report side effects in multiple categories does not total 100%

When asked if they knew which medications they believed to be causing the side effects reported in the ‘three most‐bothersome list’, participants responded ‘Yes’ in 143 instances (34.4%) of all 416 side effects included in the ranking, most commonly reporting antipsychotics such as quetiapine (*n* = 35), olanzapine (*n* = 19) and clozapine (*n* = 12), which is consistent with the most prescribed class of medications in this study. Another person was reported to be aware of over half (53.4%, *n* = 222) of the ranked side effects. Participants frequently reported that family (*n* = 118; most commonly a partner) and friends (*n* = 29) were aware, compared to healthcare professionals (*n* = 17).

### 3.4 Impacts of Side Effects and Medication Use

One‐fifth of participants (21.8%, *n* = 34) indicated that they had considered not taking their medications due to the side effects and more than a quarter (29.5%, *n* = 46) reported that they had stopped taking medications due to the impact of these side effects.

When prompted to answer why they had stopped taking medications, a content analysis of the open‐ended responses from 44 participants generated four categories: (1) impact of the side effects; (2) lack of insight into medication need; (3) stigma‐related impacts and (4) forgetful non‐adherence. The categories, including a descriptor and illustrative quote(s), are shown in Table 6. Notably, responses to this question identified reasons for cessation of medications that were not always strictly related to experiencing side effects.

**Table 6:** Reasons for stopping medications (*n* = 44).

Category	Description	Illustrative quotes
Impact of side effects	The impact of side effects, particularly relating to drowsiness or weight gain was a reason for stopping medications.	*Fed up with being fatigued/lethargic all the time.* [ID 17]
		*Because I wanted to function the next day.* [ID90]
		*Hated the fuzzy head.* [ID32]
Lack of perceived need for medication	Some consumers stopped medication when they thought they were feeling better or did not believe the medications were necessary.	*Felt in control of life at that stage, however, was not.* [ID60]
		*Wanted to see if it was doing anything.* [ID145]
		*Because I found they didn't work—a long while ago.* [ID52]
		*Because felt they were not necessary*. [ID82]
Stigma‐related impacts	Some consumers reported ceasing medication due to the stigma associated with mental illness.	*The feeling of being depressed by having the need to take medication… worried about the stigma of taking medication for* [mental illness]. [ID 87]
		*Struggled with diagnosis*… [ID 109]
Forgetful non‐adherence	Unintentional non‐adherence was reported, where consumers stated they simply forgot to take their medications.	*Forgetting to take it.* [ID137]
		*Forgetting to take quetiapine at night*. [ID123]

### 3.5 Perceived Benefits of Medication Use

The *M3Q* also prompts respondents to consider benefits of medication use. Open‐ended responses to this question were received from 89 participants. Content analysis of these responses generated four categories: (1) control and stability; (2) improved mood; (3) keeping out of hospital and (4) social and workforce participation. The categories, including a descriptor and illustrative quotes, are shown in Table 7.

**Table 7:** Consumer reported medication benefits (*n* = 89).

Category	Description	Illustrative quote/s
Control and stability	Consumers reported benefits in controlling their symptoms, being stable and feeling in control.	*I'm more in control of myself.* [ID 16]
		*More stable. Less conflicting thoughts. Less time dwelling on particular issues compared to previous times*. [ID 74]
		*Improve my sleep, stop me from feeling on edge/nervous.* [ID 17]
Improved mood	Medications were reported to help improve mood and decrease risk of suicide.	*A lot more happier. Doing a lot more at home. Not just sitting down and watching TV all the time and feeling depressed. Back going out with friends.* [ID 31]
		*Don't feel like having to commit suicide.* [ID 47]
		*Stops sadness, anxiety, social isolation.* [ID 62]
Keeping out of hospital	Some consumers reported their medications helped them keep out of hospital.	*Keeping well, happy, stable, out of hospital.* [ID 51]
Social and workforce participation	Consumers highlighted benefits they experienced from their medications including being able to participate in the workforce, have a social life and improved relationships with family and friends.	*I'm alive!… able to work and have a social life.* [ID 87]
		*Better relationships with children, feels a lot better mentally, better at work.* [ID 109]
		*Living a more stable and functioning lifestyle.* [ID 130]

## 4 Discussion

The findings of the current study contribute to the growing body of evidence regarding consumer experience of using psychotropic medications to manage mental illness. Importantly, the current study has identified the range of side effects experienced by a sample of people living with SPMI, with almost all participants reporting at least one side effect and many reporting experiencing several side effects. Sleep‐related issues, issues related to mood and appetite or weight changes were reported by consumers as being both common and bothersome.

The common incidence of reported side effects in this study highlights the lived experience of the burden of side effects of psychotropic medications. These findings further support the need for members of mental healthcare teams to engage with consumers to directly enquire about side effects of treatment, either using validated tools or other techniques. Moreover, consumers in this sample reported that family and friends were more aware of their experience of side effects than healthcare professionals. This is unsurprising and aligns with existing literature which demonstrates that consumers may be unlikely to disclose their experience of side effects unless directly asked and may turn to the Internet rather than healthcare professionals to obtain information about their psychotropic medications [23, 46]. The findings of this study demonstrate that consumers were willing to report side effects to a healthcare professional using a validated tool.

It is well‐known that experiencing side effects can negatively impact adherence to psychotropic medications [13, 47]. Consumers in this study reported either considering stopping their medications or stopping their medications as a result of side effects they experienced. Open ended responses further highlighted the impact of side effects on their daily lives, particularly the impact of feeling drowsy or experiencing a "*fuzzy head*". It is also important to note that other factors contributing to non‐adherence were also reported including stigma, a lack of insight into the need for medication and simply forgetting to take medication. Despite reporting less favourable aspects of the treatment experience, consumers also recognised the benefits of medications and particularly identified their role in symptom control, reduced suicidal ideation and avoiding hospitalisation. These findings demonstrate the important role of healthcare professionals engaging with consumers regarding both positive and negative aspects of psychotropic treatment to foster informed decision making regarding the use of medications.

Given the association between non‐adherence and the risk of poorer outcomes such as hospitalisation [12], it is critical that any concerns or misconceptions regarding medication use, including side effects, are discussed with consumers and appropriately mitigated through shared decision‐making [48]. All members of the multidisciplinary mental healthcare team may be able to support consumers in this manner. As medication experts, pharmacists can play a crucial role in identifying and addressing medication‐related concerns and providing education through processes such as structured medication reviews [49]. Previous research has identified the potential benefits of interventions where pharmacists have worked with consumers to discuss psychotropic medications and side effects. A study in Türkiye found an increase in self‐reported adherence scores following an educational medication counselling session delivered by a clinical pharmacist [29]. Similarly, a study in Japan demonstrated the benefits of pharmacist‐delivered education on side effects of psychotropic medications, finding that consumers were more likely to self‐identify side effects they were experiencing after the educational intervention [30]. Specific interventions, strategies and tools to enhance regular dialogic communication between pharmacists and consumers using psychotropic medications are needed. Such interventions and strategies could promote the early detection and appropriate management of medication‐related concerns, including side effects, to improve the treatment experience and foster adherence to medications, ultimately enhancing the recovery journey. Further to benefits to adherence, identification of side effects and their impacts for consumers is important for members of the mental healthcare team to aid treatment decisions. Several clinical practice guidelines for the management of SPMI highlight the important role of side effect profiles in selection and dosing of pharmacotherapy [3, 50, 51].

Consistent with existing literature [52, 53], sleep‐related side effects and appetite or weight changes were reported by consumers to be among the most common and bothersome side effects. A previous study using the *M3Q* demonstrated 77% of participants using psychotropic medications reported sedation and ranked weight gain as the most bothersome side effect [41]. Although relatively consistent with previous responses to the *M3Q* [41], a somewhat concerning finding was the rates of mood‐related side effects reported by the consumers in this study. Mood‐related side effects may be associated with psychotropic medications prescribed in this population, particularly, antidepressants, however, such significant rates were not anticipated. While this finding warrants further investigation and consideration, several factors may have contributed the incidence of these side effects in this study. Moderate‐severe depression and moderate‐severe anxiety were common self‐reported diagnoses in this sample, and it is possible that the experience of feeling anxious, sad or agitated may be attributed to poorly controlled illness rather than true side effects. Furthermore, these symptoms are consistent with other conditions under the SPMI umbrella, such as negative symptoms of schizophrenia and hence may have been related to SPMI rather than psychotropic medications. Furthermore, this study was conducted during peak times of the COVID‐19 pandemic when access to regular supports and face‐to‐face interactions with peers and healthcare professionals, including pharmacists, were limited and telehealth appointments became more prominent [54, 55]. This may have contributed to negative affective symptoms, noting that some clinicians reported symptomatic changes in consumers and levels of psychological distress were heightened in both people living with mental illness, including those in this study population, as well as the general population during the height of the pandemic [56, 57, 58, 59]. Ultimately, the degree of poorly controlled illness and the influence of the COVID‐19 environment on the side effects reported in this study remains unknown and further work is required to investigate this and, importantly, appropriately screen and refer consumers who may have suboptimally managed mental illness.

Furthering understanding of the lived experience of the use of psychotropic medications, such as the experience of side effects, and mental illness and privileging the lived experience voice is critical to developing interventions and services that both meet the needs of and better support people living with mental illness. The findings of this study further highlight the need for interventions such as tailored medication management and holistic support services for people living with SPMI. As medication experts, pharmacists could play a role in supporting consumers through routinely and proactively screening for side effects and working with consumers and the healthcare team to manage these side effects, in addition to wider medication management services and education and holistic health interventions which manage other critical aspects, such as lifestyle and nutrition. Further research is required to continue to build the evidence base for how best to design such interventions and to evaluate the effectiveness of these interventions.

Findings of the current study must be considered in the context of relevant strengths and limitations of both the study method and the *M3Q* tool itself. A validated tool was used in this study to elicit the side effects of psychotropic medications experienced by a sample of people living with SPMI across four Australian regions. A strength of the study includes capturing the lived experience of side effects from a group of consumers who reside in the community. While use of a validated instrument that has been purposefully designed to elicit the lived experience of psychotropic side effects is a strength of this study, some limitations must also be considered. Although the tool has undergone validation processes, the data collected is participants' self‐reporting of side effects, which may be subjective, noting that the *M3Q* was not designed to generate an objective record of side effects, but rather focus on consumers' perceptions and experiences of side effects [41]. It is unknown how pharmacist administration of the tool, rather than participant self‐completion of the tool, may have impacted findings. It is possible that participants may have demonstrated a greater degree of response bias than if the tool was self‐completed, potentially resulting in selective reporting of under‐reporting of certain side effects due to the presence of the pharmacist. Furthermore, responses to certain questions, such as the cessation of medications due to side effects, did not appear to be consistent with what the question was asking, and adverse effects commonly reported in this study such as nausea and xerostomia are notable omissions from the tool. Consequently, further testing and validation of the instrument may be warranted. Written responses to open‐ended questions were brief, limiting the possibility of in‐depth analysis and insights regarding consumers' experiences. With the available data it was not possible to determine the relationships and degree of interaction between categories identified in the content analysis, likely oversimplifying what is a complex construct. Further research using qualitative methods is needed to investigate these phenomena in greater depth. Additionally, the M3Q lacks certain response options, such as ‘not applicable’ or ‘not experiencing any side effects’ options for each item, so it is not possible to determine if missing data is truly missing or intentional non‐response where participants' responses did not align with any of the available options.

The sample included in this study may not be indicative of the broader population living with SPMI, particularly, noting the significant proportion of participants in this study who reported that they were born in Australia and speaking English at home. Further research with samples that are representative of the broader population are warranted. Finally, the list of medications reported in this study excluded medications that may be used for other mental health conditions such as attention deficit hyperactivity disorder or substance use disorder. It is also possible that some psychotropic medications included in this study were used off‐label for indications, such as chronic pain or insomnia, and not for SPMI. However, pharmacists were required to ensure that consumers had to be taking at least one psychotropic or mood stabiliser medication to participate in the RCT. Finally, much of the data collected to inform medication lists, indications for medications, and mental illness diagnoses was self‐reported, which may be subject to bias.

## 5 Conclusion

Almost all participants in this sample of consumers living with SPMI reported experiencing at least one side effect attributable to their psychotropic medications. Many consumers experienced multiple side effects with varying degrees of frequency. Sleep‐related issues, issues related to mood and appetite or weight changes were reported as both common and bothersome. These findings highlight the need to actively engage with consumers using psychotropic medications to have regular open discussions regarding their experience of using such medications, including side effects. Identifying side effects or other issues with psychotropic medications may present opportunities to work together with consumers to improve their pharmacotherapy experience and reduce the risk of non‐adherence.

## Author Contributions

**Jack C. Collins:** conceptualisation, methodology, formal analysis, investigation, data curation, writing–review and editing, writing–original draft. **Amanda J. Wheeler:** conceptualisation, methodology, investigation, writing–review and editing, funding acquisition, supervision. **Sara S. McMillan:** conceptualisation, methodology, investigation, writing–review and editing; funding acquisition. **Jie Hu:** methodology, investigation, data curation, writing–review and editing. **Sarira El‐Den:** conceptualisation, methodology, investigation, writing–review and editing; funding acquisition. **Helena Roennfeldt:** conceptualisation, methodology, investigation, writing–review and editing. **Claire L. O'Reilly:** conceptualisation, methodology, funding acquisition, investigation, formal analysis, writing–review and editing, supervision.

## Ethics Statement

The study from which this data is obtained received approval from the Griffith University Human Research Ethics Committee (HREC/2019/473).

## Consent

Participants provided informed written consent before participation in this study.

## Conflicts of Interest

The authors declare no conflicts of interest.

## Data Availability

The authors have nothing to report.
